# Deep Feature Mining *via* the Attention-Based Bidirectional Long Short Term Memory Graph Convolutional Neural Network for Human Motor Imagery Recognition

**DOI:** 10.3389/fbioe.2021.706229

**Published:** 2022-02-11

**Authors:** Yimin Hou, Shuyue Jia, Xiangmin Lun, Shu Zhang, Tao Chen, Fang Wang, Jinglei Lv

**Affiliations:** ^1^ School of Automation Engineering, Northeast Electric Power University, Jilin, China; ^2^ School of Computer Science, Northeast Electric Power University, Jilin, China; ^3^ College of Mechanical and Electric Engineering, Changchun University of Science and Technology, Changchun, China; ^4^ School of Computer Science, Northwestern Polytechnical University, Xi’an, China; ^5^ School of Biomedical Engineering and Brain and Mind Center, University of Sydney, Sydney, NSW, Australia

**Keywords:** brain–computer interface (BCI), electroencephalography (EEG), motor imagery (MI), bidirectional long short-term memory (BiLSTM), graph convolutional neural network (GCN)

## Abstract

Recognition accuracy and response time are both critically essential ahead of building the practical electroencephalography (EEG)-based brain–computer interface (BCI). However, recent approaches have compromised either the classification accuracy or the responding time. This paper presents a novel deep learning approach designed toward both remarkably accurate and responsive motor imagery (MI) recognition based on scalp EEG. Bidirectional long short-term memory (BiLSTM) with the attention mechanism is employed, and the graph convolutional neural network (GCN) promotes the decoding performance by cooperating with the topological structure of features, which are estimated from the overall data. Particularly, this method is trained and tested on the short EEG recording with only 0.4 s in length, and the result has shown effective and efficient prediction based on individual and groupwise training, with 98.81% and 94.64% accuracy, respectively, which outperformed all the state-of-the-art studies. The introduced deep feature mining approach can precisely recognize human motion intents from raw and almost-instant EEG signals, which paves the road to translate the EEG-based MI recognition to practical BCI systems*.*

## 1 Introduction

Recently, the brain–computer interface (BCI) has played a promising role in assisting and rehabilitating patients from paralysis, epilepsy, and brain injuries *via* interpreting neural activities to control the peripherals (Bouton et al., 2016; Schwemmer et al., 2018). Among the noninvasive brain activity acquisition systems, electroencephalography (EEG)-based BCI has gained extensive attention recently given its higher temporal resolution and portability. Hence, it has been popularly employed to assist the recovery of patients from motor impairments, e.g., amyotrophic lateral sclerosis (ALS), spinal cord injury (SCI), or stroke survivors (Daly and Wolpaw, 2008; Pereira et al., 2018). Specifically, researchers have focused on the recognition of motor imagery (MI) based on EEG and translating brain activities into specific motor intentions. In such a way, users can further manipulate external devices or exchange information with the surroundings (Pereira et al., 2018). Although researchers have developed several MI-based prototype applications, there is still space for improvement before the practical clinical translation could be promoted (Schwemmer et al., 2018; Mahmood et al., 2019). *De facto*, to achieve effective and efficient control *via* only MI, both precise EEG decoding and quick response are eagerly expected. However, few existing works of literature are competent in both perspectives. In this study, we explore the possibility of a deep learning framework to tackle the challenge.

### 1.1 Related Work

Lately, deep learning (DL) has attracted increasing attention in many disciplines because of its promising performance in classification tasks (LeCun et al., 2015). A growing number of works have shown that DL will play a pivotal role in the precise decoding of brain activities (Schwemmer et al., 2018). Especially, recent works have been carried out on EEG motion intention detection. A primary current focus is to implement the DL-based approach to decode EEG MI tasks, which have attained promising results (Lotte et al., 2018). Due to the high temporal resolution of EEG signals, methods related to the recurrent neural network (RNN) (Rumelhart et al., 1986), which can analyze time-series data, were extensively applied to filter and classify EEG sequences, i.e., time points (Güler et al., 2005; Wang P et al., 2018; Luo et al., 2018; Zhang T et al., 2018; Zhang X et al., 2018). In reference to Zhang T et al. (2018), a novel RNN framework with spatial and temporal filtering was put forward to classify EEG signals for emotion recognition and achieved 95.4% accuracy for three classes with a 9-s segment as a sample. Yang et al. also proposed an emotion recognition method using long short-term memory (LSTM) (Yang J et al., 2020). Wang et al. and Luo et al. performed LSTM (Hochreiter and Schmidhuber, 1997) to handle signals of time slices and achieved 77.30% and 82.75% accuracy, respectively (Wang P et al., 2018; Luo et al., 2018). Zhang X et al. (2018) presented attention-based RNN for EEG-based person identification, which attained 99.89% accuracy for eight participants at the subject level with 4-s signals as a sample. LSTM was also employed in some medical fields, such as seizure detection (Hu et al., 2020), with the recorded EEG signals. However, it can be found that in these studies, signals over experimental duration were recognized as samples, which resulted in a slow responsive prediction.

Apart from RNN, the convolutional neural network (CNN) (Fukushima, 1980; LeCun et al., 1998) has been performed to decode EEG signals as well (Dose et al., 2018; Hou et al., 2020). Hou et al. proposed ESI and CNN and achieved competitive results, i.e., 94.50% and 96.00% accuracy at the group and subject levels, respectively, for four-class classification. What is more, by combining CNN with the graph theory, the graph convolutional neural network (GCN) (Bruna et al., 2014; Henaff et al., 2015; Duvenaud et al., 2015; Niepert et al., 2016; Defferrard et al., 2016) approach was presented lately, taking consideration of the functional topological relationship of EEG electrodes (Wang XH et al., 2018; Song et al., 2018; Zhang T et al., 2019; Wang et al., 2019). In reference to Wang XH et al. (2018) and Zhang T et al. (2019), a GCN with a broad learning approach was proposed and attained 93.66% and 94.24% accuracy, separately, for EEG emotion recognition. Song et al. and Wang et al. introduced dynamical GCN (90.40% accuracy) and phase-locking value-based GCN (84.35% accuracy) to recognize different emotions (Song et al., 2018; Wang et al., 2019). A highly accurate prediction has been accomplished *via* the GCN model. Few researchers have investigated the approach in the area of EEG MI decoding.

### 1.2 Contribution of This Paper

Toward accurate and fast MI recognition, an attention-based BiLSTM–GCN was introduced to mine the effective features from raw EEG signals. The main contributions were summarized as follows:

 i) As far as we know, this work was the first that combined BiLSTM with the GCN to decode EEG tasks.
 ii) The attention-based BiLSTM successfully derived relevant features from raw EEG signals. Followed by the GCN model, it enhanced the decoding performance by considering the internal topological structure of features.
iii) The proposed feature mining approach managed to decode EEG MI signals with stably reproducible results yielding remarkable robustness and adaptability that deals with the considerable intertrial and intersubject variability.

### 1.3 Organization of This Paper

The rest of this paper was organized as follows. The preliminary knowledge of the BiLSTM, attention mechanism, and GCN was introduced in the *Methodology* section, which was the foundation of the presented approach. In the *Results and Discussion* section, experimental details and numerical results were presented, followed by the conclusion in the *Conclusion* section.

## 2 Methodology

### 2.1 Pipeline Overview

**FIGURE 1:**
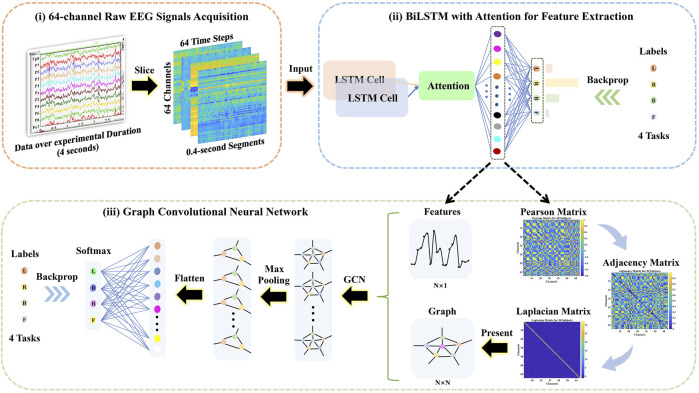
The schematical overview consisted of the 64-channel raw electroencephalography (EEG) signal acquisition, the bidirectional long short-term memory (BiLSTM) with the attention model for feature extraction, and the graph convolutional neural network (GCN) model for classification.

The framework of the proposed method is presented in Figure 1.

 i) The 64-channel raw EEG signals were acquired *via* the BCI 2000, and then the 4-s (experimental duration) signals were sliced into 0.4-s segments over time, where the dimension of each segment was 64 channels × 64 time steps.
 ii) The attention-based BiLSTM was put forward to filter 64-channel (spatial information) and 0.4-s (temporal information) raw EEG data and derived features from the fully connected neurons.
iii) The Pearson, adjacency, and Laplacian matrices of overall features were introduced sequentially to represent the topological structure of features, i.e., as a graph. Followed by the features and its corresponding graph representation as the input, the GCN model was performed to classify four-class MI tasks.

### 2.2 Bidirectional Long Short Term Memory With Attention

#### 2.2.1 Bidirectional Long Short Term Memory Model

**FIGURE 2:**
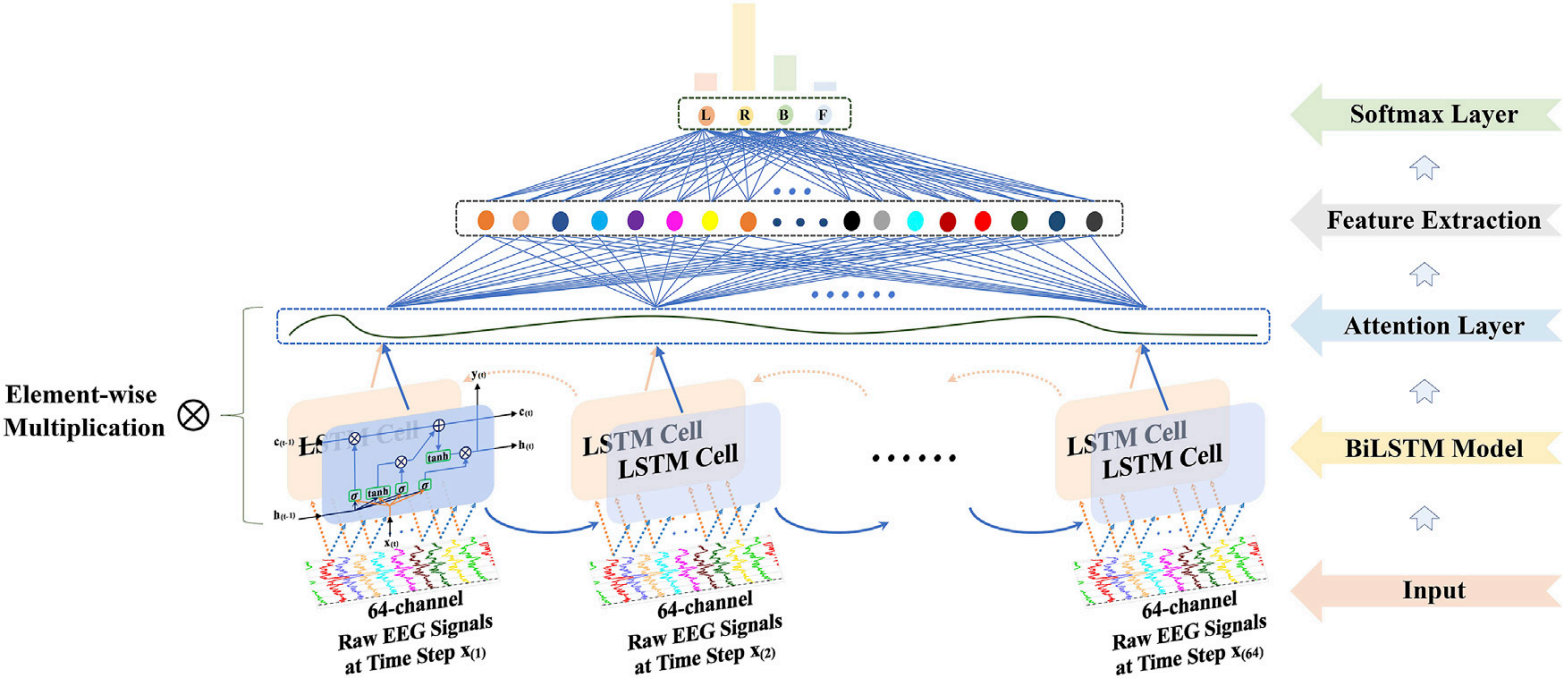
Presented BiLSTM with the attention mechanism for feature extraction.

RNN-based approaches have been extensively applied to analyze EEG time-series signals. An RNN cell, though alike a feedforward neural network, has connections pointing backward, which sends its output back to itself. The learned features of an RNN cell at time step *t* are influenced by not only the input signals **x**_(*t*)_ but also the output (state) at time step *t* − 1. This design mechanism dictates that RNN-based methods can handle sequential data, e.g., time point signals, by unrolling the network through time. The LSTM and gated recurrent unit (GRU) (Cho et al., 2014) are the most popular variants of the RNN-based approaches. In the*Proposed approach*section, the paper compared the performance of the welcomed models experimentally, and the BiLSTM with attention displayed in Figure 2 outperformed others due to better detection of the long-term dependencies of raw EEG signals.

$$i_{\left(t\right)}=\sigma \left(W_{xi}^{T}\cdot x_{\left(t\right)}+W_{hi}^{T}\cdot h_{\left(t-1\right)}+b_{i}\right)$$ (1)

$$f_{\left(t\right)}=\sigma \left(W_{xf}^{T}\cdot x_{\left(t\right)}+W_{hf}^{T}\cdot h_{\left(t-1\right)}+b_{f}\right)$$ (2)

$$o_{\left(t\right)}=\sigma \left(W_{xo}^{T}\cdot x_{\left(t\right)}+W_{ho}^{T}\cdot h_{\left(t-1\right)}+b_{o}\right)$$ (3)

$$g_{\left(t\right)}=\tanh\left(W_{xg}^{T}\cdot x_{\left(t\right)}+W_{hg}^{T}\cdot h_{\left(t-1\right)}+b_{g}\right)$$ (4)

$$c_{\left(t\right)}=f_{\left(t\right)}⊗c_{\left(t-1\right)}+i_{\left(t\right)}⊗g_{\left(t\right)}$$ (5)

$$y_{\left(t\right)}=h_{\left(t\right)}=o_{\left(t\right)}⊗\tanh\left(c_{\left(t\right)}\right)$$ (6)

As illustrated in Figure 2, three kinds of gates manipulate and control the memories of EEG signals, namely, the input gate, forget gate, and output gate. Demonstrated by the **i**_(*t*)_, the input gate partially stores the information of **x**_(*t*)_ and controls which part of it should be added to the long-term state **c**_(*t*)_. The forget gate controlled by the **f**_(*t*)_ decides which piece of the **c**_(*t*)_ should be overlooked. The output gate, controlled by **o**_(*t*)_, allows which part of the information from **c**_(*t*)_ should be outputted, denoted as **y**_(*t*)_, known as the short-term state **h**_(*t*)_. Manipulated by the above gates, two kinds of states are stored. The long-term state **c**_(*t*)_ travels through the cell from left to right, dropping some memories at the forget gate and adding something new from the input gate. After that, the information passes through a nonlinear activation function, tanh activation function usually, and then it is filtered by the output gate. In such a way, the short-term state **h**_(*t*)_ is produced.

Eqs. 1–6 describe the procedure of an LSTM cell, where **W** and **b** are the weights and biases for different layers to store the memory and learn a generalized model, and *σ* is a nonlinear activation function, i.e., sigmoid function used in the experiments. For bidirectional LSTM, BiLSTM for short, the signals **x**_(*t*)_ are inputted from left to right for the forward LSTM cell. What is more, they are reversed and inputted into another LSTM cell, the backward LSTM. Thus, there are two output vectors, which store much more comprehensive information than a single LSTM cell. Then they are concatenated as the final output of the cell.

#### 2.2.2 Attention Mechanism

The attention mechanism, imitated from the human vision, has a vital part to play in the field of computer vision (CV), natural language processing (NLP), and automatic speech recognition (ASR) (Bahdanau et al., 2014; Chorowski et al., 2015; Xu et al., 2015; Yang et al., 2016). Not all the signals contribute equally toward the classification. Hence, an attention mechanism **s**_(*t*)_ is jointly trained as a weighted sum of the output of the BiLSTM with attention based on the weights.

$$u_{\left(t\right)}=\tanh\left(W_{w}y_{\left(t\right)}+b_{w}\right)$$ (7)

$$\alpha_{\left(t\right)}=\frac{\exp\left(u_{\left(t\right)}^{⊤}u_{w}\right)}{\sum_{t}\exp\left(u_{\left(t\right)}^{⊤}u_{w}\right)}$$ (8)

$$s_{\left(t\right)}=\underset{t}{\sum }\alpha_{\left(t\right)}y_{\left(t\right)}$$ (9)

**u**_(*t*)_ is a fully connected (FC) layer for learning features of **y**_(*t*)_, followed by a softmax layer which outputs a probability distribution ***α***_(*t*)_. **W**_*w*_, **u**_*w*_, and **b**_*w*_ denote trainable weights and biases, respectively. It selects and extracts the most significant temporal and spatial information from **y**_(*t*)_ by multiplying ***α***_(*t*)_ with regard to the contribution to the decoding tasks.

### 2.3 Graph Convolutional Neural Network

#### 2.3.1 Graph Convolution

In the graph theory, a graph is presented by the graph Laplacian *L*. It is computed by the degree matrix *D* minus the adjacency matrix *A*, i.e., *L* = *D* − *A*. In this work, Pearson’s matrix *P* was utilized to measure the inner correlations among features.

$$P_{X,Y}=\frac{E\left(\left(X-\mu_{X}\right)\left(Y-\mu_{Y}\right)\right)}{\sigma_{X}\sigma_{Y}}$$ (10)

where *X* and *Y* are two variables regarding different features, *ρ*_*X*,*Y*_ is their correlation, *σ*_*X*_ and *σ*_*Y*_ are the standard deviations, and *μ*_*X*_ and *μ*_*Y*_ are the expectations. Besides, the adjacency matrix *A* is recognized as:

A=|P|−I (11)

where |*P*| is the absolute of Pearson’s matrix *P*, and $I\in R^{N\times N}$ is an identity matrix. In addition, the degree matrix *D* of the graph is computed as follows:

$$D_{ii}=\overset{N}{\underset{j=1}{\sum }}A_{ij}$$ (12)

Then the normalized graph Laplacian is computed as:

$$L=D-A=I_{N}-D^{-1/2}AD^{-1/2}$$ (13)

It is then decomposed by the Fourier basis $U=\left[u_{0},\ldots ,u_{N-1}\right]\in R^{N\times N}$ . The graph Laplacian is described as *L* = *U*Λ*U* ^*T*^, where $\Lambda =\mathrm{diag}\left(\left[\lambda_{0},\ldots ,\lambda_{N-1}\right]\right)\in R^{N\times N}$ are the eigenvalues of *L*. The graph convolution is defined as:

$$y=g_{\theta }\left(L\right)x=g_{\theta }\left(U\Lambda U^{T}\right)x=Ug_{\theta }\left(\Lambda \right)U^{T}x$$ (14)

in which *g*_*θ*_ is a nonparametric filter. Specifically, the operation is as follows:

$$y_{:,j}^{k}=\sigma \left(\overset{f_{k-1}}{\underset{i=1}{\sum }}Ug_{\theta }\left(\Lambda \right)U^{T}x_{:,i}^{k-1}\right)$$ (15)

in which $x^{k-1}\in R^{N\times f_{k-1}}$ denotes the signals, *N* is the number of vertices of the graph, *f*_*k*−1_ and *f*_*k*_ are the numbers of input and output channels, respectively, and *σ* denotes a nonlinearity activation function. What is more, *g*_*θ*_ is approximated by the Chebyshev polynomials because it is not localized in space and very time-consuming (Hammond et al., 2011). The Chebyshev recurrent polynomial approximation is described as *T*_*k*_(*x*) = 2*xT*_*k*−1_(*x*) − *T*_*k*−2_(*x*), *T*_0_ = 1, *T*_1_ = *x*. The filter can be presented as $g_{\theta }\left(\Lambda \right)=\sum_{k=0}^{K-1}\theta_{k}T_{k}\left(\tilde{\Lambda }\right)$ , in which $\theta \in R^{K}$ is a set of coefficients, and $T_{k}\left(\tilde{\Lambda }\right)\in R^{K}$ is the *k*th-order polynomial at $\tilde{\Lambda }=2\Lambda /\lambda_{max}-I_{n}$ , and *I*_*n*_ ∈ (−1, 1) is a diagonal matrix of the scaled eigenvalues. The convolution can be rewritten as:

$$y=\overset{K-1}{\underset{k=0}{\sum }}\theta_{k}L^{k}x$$ (16)

#### 2.3.2 Graph Pooling

The graph pooling operation can be achieved *via* the Graclus multilevel clustering algorithm, which consists of node clustering and one-dimensional pooling (Dhillon et al., 2007). A greedy algorithm was implemented to compute the successive coarser of a graph and minimize the clustering objective, from which the normalized cut was chosen (Shi and Malik, 2000). Through such a way, meaningful neighborhoods on graphs were acquired. Defferrard et al. (2016) proposed to carry out a balanced binary tree to store the neighborhoods, and a one-dimensional pooling was then applied for precise dimensionality reduction.

### 2.4 Proposed Approach

**FIGURE 3:**
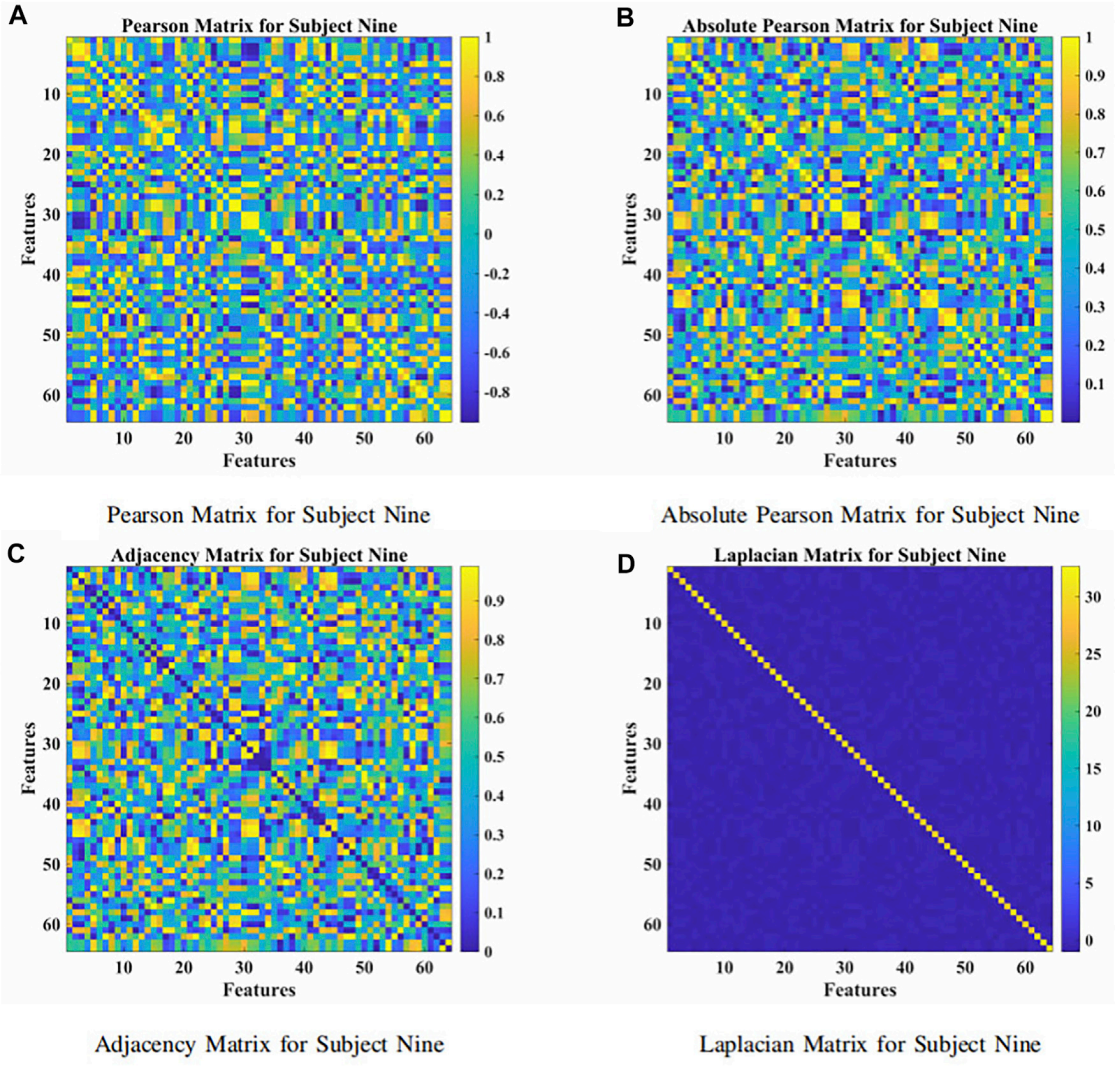
The Pearson, absolute Pearson, adjacency, and Laplacian matrices for subject nine. **(A)** Pearson matrix for subject nine. **(B)** Absolute Pearson matrix for subject nine. **(C)** Adjacency matrix for subject nine. **(D)** Laplacian matrix for subject nine.

The presented approach was a combination of the attention-based BiLSTM and the GCN, as illustrated in Figure 1. The BiLSTM with the attention mechanism was presented to derive relevant features from raw EEG signals. During the procedure, features were obtained from neurons at the FC layer. In Figure 3, we demonstrated the topological connections of the Subject Nine’s features via the Pearson Matrix, Absolute Pearson Matrix, Adjacency Matrix, and Laplacian Matrix. The GCN was then applied to classify the extracted features. It was the combination of two models that promoted and enhanced the decoding performance by a significant margin compared with existing studies. Details were provided in the following.

**FIGURE 4:**
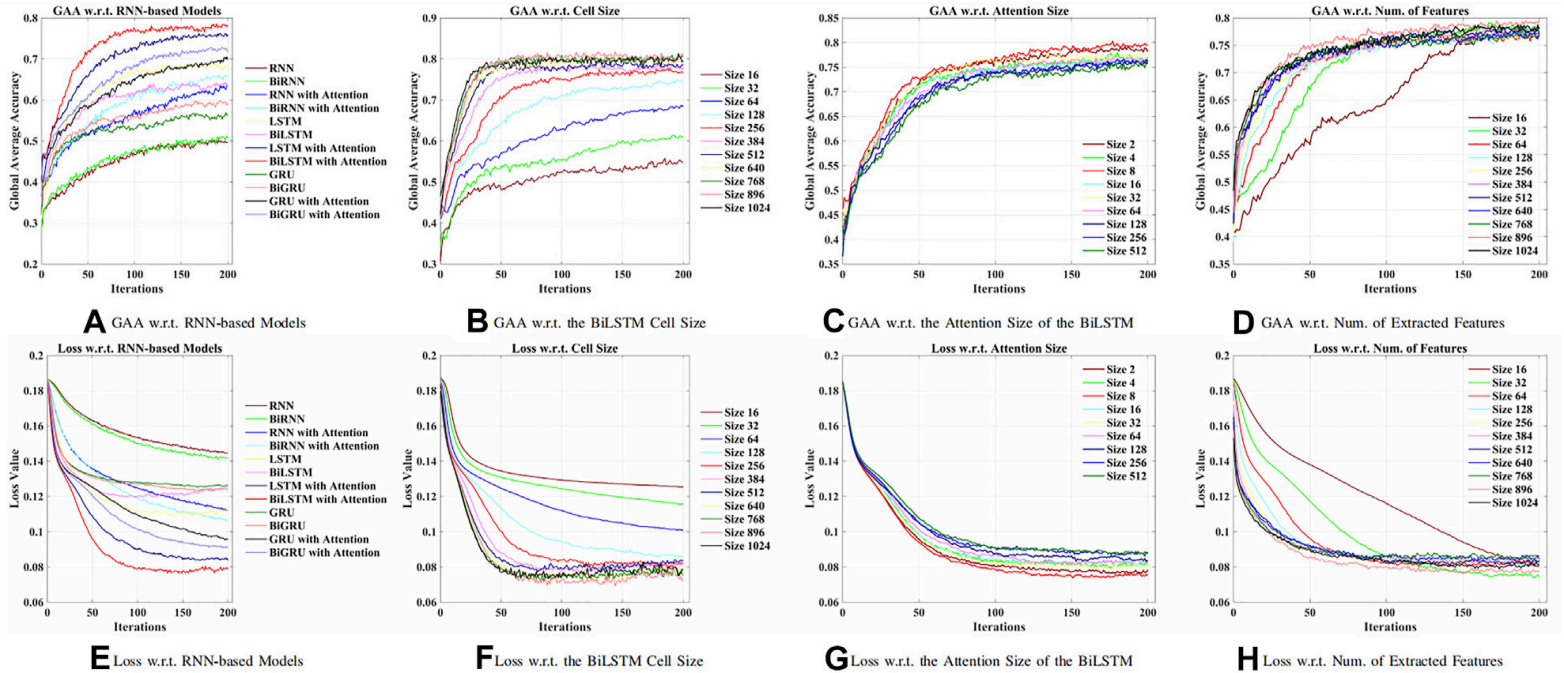
Comparison of models and hyperparameters w.r.t. the recurrent neural network (RNN)-based methods for feature extraction. **(A)** Global average accuracy (GAA) w.r.t. RNN-based models. **(B)** GAA w.r.t. BiLSTM cell size. **(C)** GAA w.r.t. attention size of the BiLSTM. **(D)** GAA w.r.t. the number of the extracted features. **(E)** Loss w.r.t. RNN-based models. **(F)** Loss w.r.t. BiLSTM cell size. **(G)** Loss w.r.t. attention size of the BiLSTM. **(H)** Loss w.r.t. the number of the extracted features.

First of all, an optimal RNN-based model was explored to obtain relevant features from raw EEG signals. As shown in Figure 4, in this work, the BiLSTM with the attention model was best performed, which achieved 77.86% global average accuracy (GAA). The input size **x**_(*t*)_ of the model was 64, denoting 64 channels (electrodes) of raw EEG signals. The maximum time *t* was chosen as 64, which was a 0.4-s segment. According to Figures 4A, B, higher accuracy has been obtained while increasing the number of cells of the BiLSTM model. It should, however, be noted in Figure 3F that when there were more than 256 cells, the loss showed an upward trend, which indicated the concern of overfitting due to the increment of the model complexity. As a result, 256 LSTM cells (76.67% GAA) were chosen to generalize the model. Meanwhile, it was apparent that, in Figure 4C, as for the linear size of the attention weights, the majority of the choices did not make a difference. Thus, eight neurons, with 79.40% GAA, were applied during the experiments empirically. Comparing Figures 4D, H, it showed that a compromise solution should be applied, which took into consideration both performance and input size of the GCN. As a result, a linear size of 64 (76.73% GAA) was utilized at the FC layer.

Besides, to prevent overfitting, a 25% dropout (Srivastava et al., 2014) for the BiLSTM and FC layer was implemented. The model carried out batch normalization (BN) (Ioffe and Szegedy, 2015) for the FC layer, which was activated by the softplus function (Hahnloser et al., 2000). The L2 norm with the 1 × 10^−7^ coefficient was applied to the Euclidean distance as the loss function. A total of 1,024 batch sizes were used to maximize the usage of GPU resources. The 1 × 10^−4^ learning rate was applied to the Adam optimizer (Kingma and Ba, 2014).

Furthermore, the second-order Chebyshev polynomial was applied to approximate convolutional filters in the experiments. The GCN consisted of six graph convolutional layers with 16, 32, 64, 128, 256, and 512 filters, respectively, each followed by a graph max-pooling layer, and a softmax layer derived the final prediction.

In addition, for the GCN model, before the nonlinear softplus activation function, BN was utilized at all of the layers except the final softmax. The 1 × 10^−7^ L2 norm was added to the loss function, which was a cross-entropy loss. Stochastic gradient descent (Zhang, 2004) with 16 batch sizes was optimized by the Adam (1 × 10^−7^ learning rate).

All the experiments above were performed and implemented by the Google TensorFlow (Abadi et al., 2016) 1.14.0 under NVIDIA RTX 2080ti and CUDA10.0.

## 3 Results and Discussion

### 3.1 Description of the Dataset

The data collected from the EEG Motor Movement/Imagery Dataset (Goldberger et al., 2000) was employed in this study. Numerous EEG trials were acquired from 109 participants performing four MI tasks, i.e., imagining the left fist (L), the right fist (R), both fists (B), and both feet (F) (21 trials per task). Each trial is a 4-s experiment duration (160 Hz sample rate) with one single task (Hou et al., 2020). In this work, a 0.4-s temporal segment of 64 channel signals, i.e., 64 channels × 64 time points, was regarded as a sample. In the *Groupwise prediction* section, we used a group of 20 subject data (*S*_1_ − *S*_20_) to train and validate our method. The 10-fold cross-validation was carried out. Further, 50 subjects (*S*_1_ − *S*_50_) were selected to verify the repeatability and stability of our approach. In the *Subject-specific adaptation* section, the dataset of individual subjects (*S*_1_ − *S*_10_) was utilized to perform subject-level adaptation. For all the experiments, the dataset was randomly divided into 10 parts, where 90% was the training set, and the remaining 10% was regarded as the test set. In the *Groupwise prediction* section, the above procedure has been carried out 10 times. Thus, it left us 10 results of 10-fold cross-validation.

### 3.2 Groupwise Prediction

**FIGURE 5:**
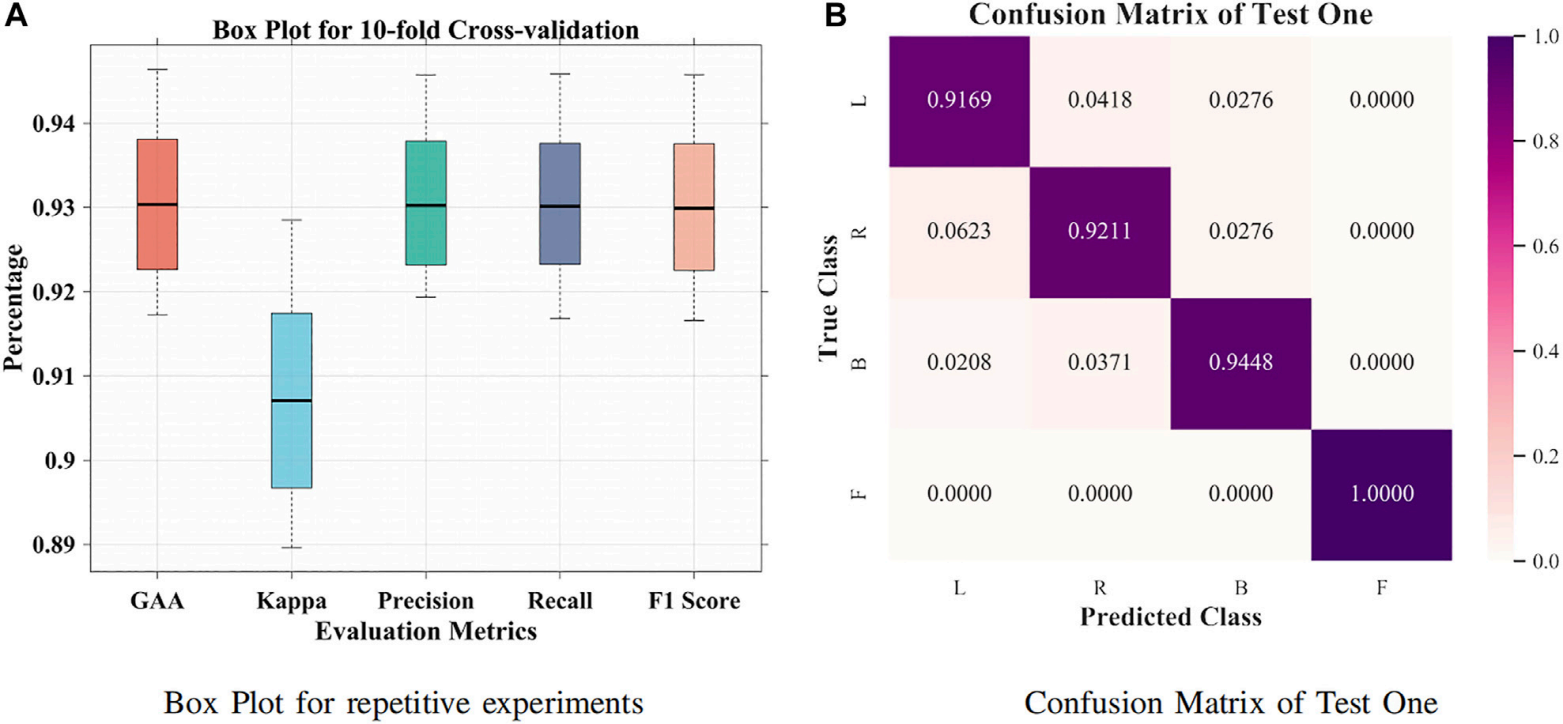
Box plot and confusion matrix for 10-fold cross-validation. **(A)** Box plot for repetitive experiments. **(B)** Confusion matrix for test one.

It was suggested that intersubject variability remains one of the concerns for interpreting EEG signals (Tanaka, 2020). First, a small group size (20 subjects) was adopted for groupwise prediction. In Figure 4A, 63.57% GAA was achieved by the BiLSTM model. After applying the attention mechanism, it enhanced the decoding performance, which accomplished 77.86% GAA (14.29% improvement). Further, we employed an attention-based BiLSTM–GCN model in this work. It attained 94.64% maximum GAA (Hou et al., 2020) (31.07% improvement compared with the BiLSTM model) and 93.04% median accuracy from 10-fold cross-validation. Our method promoted the classification capability under subject variability and variations by taking the topological relationship of relevant features into consideration. Meanwhile, as illustrated in Figure 5A, the median values of GAA, kappa, precision, recall, and F1 score were 93.04%, 90.71%, 93.02%, 93.01%, and 92.99%, respectively. To the knowledge of the authors, the proposed method has achieved the best state-of-the-art performance in group-level prediction. Besides, remarkable results of 10-fold cross-validation have verified the repeatability and stability. Furthermore, the confusion matrix of test one (94.64% GAA) was given in Figure 5B. Accuracies of 91.69%, 92.11%, 94.48%, and 100% were obtained for each task. It can be observed that our method was unprecedentedly effective and efficient in detecting human motion intents from raw EEG signals.

**FIGURE 6:**
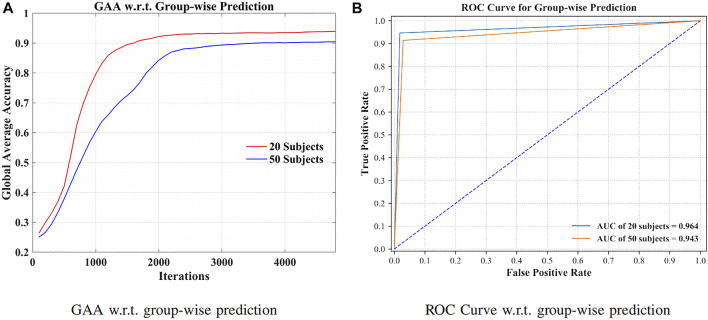
GAA and receiver operating characteristic curve (ROC curve) for 20 and 50 subjects, separately. **(A)** GAA w.r.t. groupwise prediction. **(B)** ROC curve w.r.t. groupwise prediction.

By grouping signals from additional 30 subjects (in total 50 subjects), the robustness of the method has been validated in Figure 6.

Toward practical EEG-based BCI applications, it is essential to develop a robust model to counter serious individual variability (Tanaka, 2020). Figure 6A illustrates the GAA of our method through iterations. As listed in Figure 6B, we can see that 94.64% and 91.40% GAA were obtained with regard to the group of 20 and 50 subjects, respectively. The area under the curves (AUCs) were 0.964 and 0.943. Indicated by the above results, the presented approach can successfully filter the distinctions of signals, even though the dataset was extended. In other words, by increasing the intersubject variability, the robustness and effectiveness of the method were evaluated.

The comparison of groupwise evaluation was demonstrated, measured by the maximum of GAA (Hou et al., 2020) during experiments (Ma et al., 2018; Hou et al., 2020). Here, we compared the performance of several state-of-the-art methods in Table 1.

**TABLE 1:** Comparison on groupwise evaluation.

Related work	Max. global average accuracy (GAA) (%)	Approach	Number of subjects	Database
Ma et al. (2018)	68.20	Recurrent neural networks (RNNs)	12	PhysioNet database
Hou et al. (2020)	94.50	ESI + convolutional neural networks (CNNs)	10	
	92.50		14	
This work	94.64	Attention-based bidirectional long short-term memory (BiLSTM)–graph convolutional neural network (GCN)	20	

Table 1 lists the performance of related methods. Hou et al. achieved competitive results. However, our method obtained higher performance (0.14% accuracy improvement) even with doubling the number of subjects. It can be found that our approach has outperformed those by giving the highest accuracy of decoding EEG MI signals.

### 3.3 Subject-Specific Adaptation

The performance of individual adaptation has witnessed a flourishing increment (Dose et al., 2018; Amin et al., 2019; Zhang R et al., 2019; Ji et al., 2019; Ortiz-Echeverri et al., 2019; Sadiq et al., 2019; Taran and Bajaj, 2019; Hou et al., 2020). The results of our method on subject-level adaptation have been reviewed in Table 2, and we compared the results in Table 3.

**TABLE 2:** Subject-level evaluation.

No. of subject	GAA (%)	Kappa (%)	Precision (%)	Recall (%)	F1 score (%)
1	94.05	92.06	94.20	94.32	94.16
2	96.43	95.19	96.06	96.06	96.06
3	97.62	96.79	97.33	97.08	97.18
4	90.48	87.34	91.30	91.11	90.42
5	95.24	93.61	95.96	95.06	95.38
6	94.05	92.02	93.40	94.96	93.66
7	98.81	98.40	98.81	99.07	98.92
8	95.24	93.60	95.39	95.04	95.19
9	98.81	98.39	99.11	98.68	98.87
10	94.05	91.98	93.39	94.70	93.61
Average	95.48	93.94	95.50	95.61	95.35

**TABLE 3:** Comparison of current studies on subject-level prediction.

Related work	Max. GAA (%)	Approach	Database
Ortiz-Echeverri et al. (2019)	94.66	Sorted-fast ICA-CWT + CNNs	Brain–computer interface (BCI) Competition IV-a dataset
Sadiq et al. (2019)	95.20	EWT + LS-SVM	
Taran and Bajaj (2019)	96.89	TQWT + LS-SVM	
Zhang R. et al. (2019)	83.00	CNNs–long short-term memory (LSTM)	BCI Competition IV-2a dataset
Ji et al. (2019)	95.10	SVM	
Amin et al. (2019)	95.40	MCNNs	
Dose et al. (2018)	68.51	CNNs	PhysioNet database
Hou et al. (2019)	96.00	ESI + CNNs	
This work	98.81	Attention-based BiLSTM–GCN	

Results are given in Table 2, from which the highest GAA was 98.81% achieved by subjects *S*_7_ and *S*_9_, and the lowest was 90.48% by *S*_4_. On average, the presented approach can handle the challenge of subject-specific adaptation. It achieved competitive results, with an average accuracy of 95.48%. Moreover, Cohen’s kappa coefficient (kappa), precision, recall, and F1 score were 93.94%, 95.50%, 95.61%, and 95.35%, respectively. The promising results above indicated that the introduced method filtered raw EEG signals and succeeded in classifying MI tasks.

**FIGURE 7:**
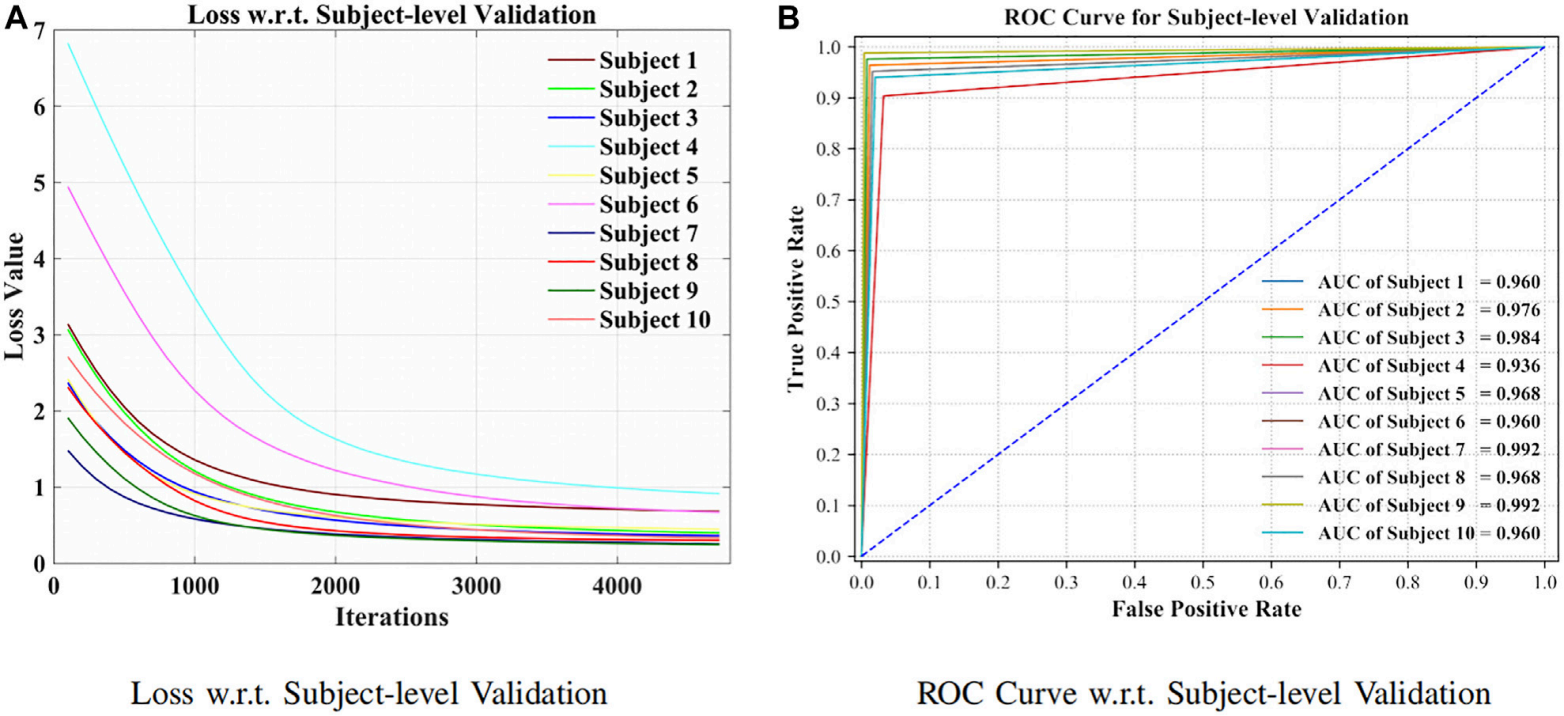
Loss and ROC curve for subject-level evaluation. **(A)** Loss w.r.t. subject-level validation. **(B)** ROC curve w.r.t. subject-level validation.

As can be seen from Figure 7A, the model has been shown to converge for the subject-specific adaptation. The receiver operating characteristic curve (ROC curve) with its corresponding AUC is visible in Figure 7B.

The comparison of subject-level prediction was put forward between the presented approach and the competitive models (Dose et al., 2018; Amin et al., 2019; Zhang R et al., 2019; Ji et al., 2019; Ortiz-Echeverri et al., 2019; Sadiq et al., 2019; Taran and Bajaj, 2019; Hou et al., 2020). The attention-based BiLSTM–GCN approach has achieved highly accurate results, which suggested robustness and effectiveness toward EEG signal processing, as shown in Table 3.

The presented approach has improved classification accuracy and obtained state-of-the-art results. The reason for the outstanding performance was that the attention-based BiLSTM model managed to extract relevant features from raw EEG signals. The followed GCN model successfully classified features by cooperating with the topological relationship of overall features.

## 4 Conclusion

To address the challenge of intertrial and intersubject variability in EEG signals, an innovative approach of attention-based BiLSTM–GCN was proposed to accurately classify four-class EEG MI tasks, i.e., imagining the left fist, the right fist, both fists, and both feet. First of all, the BiLSTM with the attention model succeeded in extracting relevant features from raw EEG signals. The followed GCN model intensified the decoding performance by cooperating with the internal topological relationship of relevant features, which were estimated from Pearson’s matrix of the overall features. Besides, results provided compelling evidence that the method has converged to both the subject-level and groupwise predictions and achieved the best state-of-the-art performance, i.e., 98.81% and 94.64% accuracy, respectively, for handling individual variability, which were far ahead of existing studies. The 0.4-s sample size was proven effective and efficient in prediction compared with the traditional 4-s trial length, which means that our proposed framework can provide a time-resolved solution toward fast response. Results on a group of 20 subjects were derived by 10-fold cross-validation, indicating repeatability and stability. The proposed method is predicted to advance the clinical translation of the EEG MI-based BCI technology to meet the diverse demands, such as of paralyzed patients. In summary, the unprecedented performance with the highest accuracy and time-resolved prediction were fulfilled *via* the introduced feature mining approach.

In addition, the proposed method in this paper could be potentially applied in relevant practical directions, such as digital neuromorphic computing to assist movement disorder (Yang et al., 2018; Yang et al., 2019; Yang S et al., 2020; Yang et al., 2021).

## Data Availability

Publicly available datasets were analyzed in this study. This data can be found here: https://www.physionet.org/content/eegmmidb/1.0.0/.
